# Role of Transient Receptor Potential Vanilloid 1 in Inflammation and Autoimmune Diseases

**DOI:** 10.3390/ph5080837

**Published:** 2012-08-17

**Authors:** Fumio Tsuji, Hiroyuki Aono

**Affiliations:** Research and Development Center, Santen Pharmaceutical Co., Ltd., 8916-16 Takayama-cho, Ikoma, Nara 630-0101, Japan; Email: aonoh@santen.co.jp (H.A.)

**Keywords:** SA13353, transient receptor potential vanilloid 1, kidney injury, lung inflammation, autoimmune diseases, pain

## Abstract

Transient receptor potential vanilloid 1 (TRPV1), a non-selective cation channel, is a receptor activated by high temperatures and chemical agonists such as the vanilloids and protons. Because of these properties, TRPV1 has emerged as a polymodal nocisensor of nociceptive afferent neurons. TRPV1 is thought to be a central transducer of hyperalgesia and a prime target for controlling pain pharmacologically because it is a point where many proalgesic pathways converge and it is upregulated and sensitized by inflammation and injury. However, whether TRPV1 agonists promote or inhibit inflammation remains unclear. We recently demonstrated that SA13353 (1-[2-(1-adamantyl)ethyl]-1-pentyl-3-[3-(4-pyridyl)propyl]urea), a novel TRPV1 agonist, inhibits tumor necrosis factor-a production by the activation of capsaicin-sensitive afferent neurons and reduces the severity of symptoms in kidney injury, lung inflammation, arthritis, and encephalomyelitis. These results suggest that TRPV1 agonists may act as anti-inflammatories in certain inflammatory and autoimmune conditions *in vivo*. Given the potential deleterious effects of inhibiting the population of channels with a protective function, caution should be taken in the use of potent TRPV1 antagonists as a general strategy to treat inflammation. Further studies are required to clarify the role of TRPV1 and neuropeptides, which are released because of TRPV1 activation in inflammation and autoimmune diseases.

## 1. Introduction

Voltage-dependent Ca^2+^ channels are well known for causing Ca^2+^ influx into the cell. A family of closely related cation channels, known as transient receptor potential (TRP) channels, has been discovered since the 1990s. These channels act as molecular sensors for distinct pain, temperature, chemesthesis, and taste modalities. The involvement of TRP channels in pain and sensing was first heralded in 1997, when vanilloid receptor 1 (renamed later TRP vanilloid 1; TRPV1) was identified at the genetic and functional level [1]. TRPV1 has a wide tissue distribution. High expression levels are observed in dorsal root ganglia, trigeminal ganglia, and nodose ganglia [1]. TRPV1 is predominantly expressed in neurons with small and medium diameters (mainly peptidergic neurons; *C*-fibers) that are important in the development of neurogenic pain and inflammation [2] and to a lesser extent in non-peptidergic neurons (Aδ-fibers) that play a critical role in mediating chronic [3] and mechanical pain [4]. Although TRPV1 distribution in the central nervous system remains controversial [5], several studies have demonstrated the expression of this channel in diverse brain regions, including the hypothalamus, cerebellum, cerebral cortex, striatum, midbrain, olfactory bulb, medulla, hippocampus, thalamus, and substantia nigra [6,7]. TRPV1 expression has also been detected in non-neuronal tissues, where its function remains unknown. Since the initial observations that stimulation of sensory neurons produces vasodilation, plasma extravasation, and hypersensitivity, much progress has been made in understanding the etiology of neurogenic inflammation [2]. TRPV1 activation leads to the release of neuropeptides, such as substance P (SP), calcitonin gene-related peptides (CGRP), and somatostatin [8]. Studies have focused largely on the role of neuropeptides, which are released in periphery from small diameter sensory *C*-fiber neuron by activation of TRPV1 in neurogenic inflammation [9,10]. Analysis of the molecular and functional properties of TRPV1 has shown that this ion channel is a polymodal nocisensor, which is subject to allosteric modulation by many proalgesic pathways. This property and its ability to become sensitized by proinflammatory mediators have raised enormous interest in TRPV1 as a prime transducer of pathological pain. Recent developments in TRPV1 agonist-based therapies primarily inactivate nociceptive nerve fibers [11]. Instillations of high-concentration of capsaicin and resiniferatoxin solutions have been found to be useful for the management of persistent bladder pain or overreactive bladder. At the same time, however, TRPV1 has been reported to have important functions in homeostasis, for example, in thermoregulation [12]. In addition, previous studies have demonstrated that neuropeptides such as CGRP [13,14,15] and somatostatin [16] inhibit lipopolysaccharide (LPS)-induced TNF-a production and modified LPS-induced other cytokines *in vivo* and *in vitro*. Furthermore, somatostatin displays anti-inflammatory and antinociceptive properties [17]. The role of TRPV1 in inflammation and autoimmune diseases is controversial; several studies have demonstrated a proinflammatory effect [18,19], while others have identified a protective role of TRPV1 in systemic inflammation and sepsis [20,21,22].

## 2. TRPV1 Structure and Modes of Action

TRPV1 is an 838-amino acid protein consisting of six transmembrane segments, with an amphipathic pore-forming region between the fifth and sixth transmembrane segments, a large *N*-terminus intracellular domain, and a *C*-terminal cytosolic region [23]. The 432-amino acid *N* terminus contains ankyrin repeat domains [24], which are essential for channel function [25] and for orchestrating a plethora of protein-protein interactions that govern the assembly of TRPV1-containing signalplexes [26,27]. The 145-amino acid *C*-terminus contains subdomains involved in distinct channel functions [28], the molecular determinants for subunit tetramerization [29], two nucleotide-binding Walker-type sites [30], and consensus sequences for modulation by phosphoinositides and protein kinases [31,32].

TRPV1 is a polymodal channel, activated by physical and chemical stimuli, including heat, vanilloids, lipids, spider toxins, protons, cations, and electrical current [33,34,35]. Intensive studies of thermoTRP channels have indicated numerous factors contributing to temperature-dependent activation. Exchanging the intracellular *C* terminus between TRPV1 and the cold-activated TRPM8 channel causes a switch in the sensitivity of thermoTRP channels to heat [36]. Cooling induced a leftward shift of the voltage activation curve of *C* terminal of cold receptor TRPM8 attached to TRPV1; the half-activation voltage decreased on cooling from 35 to 15 °C. This shift in the voltage dependence of activation agrees reasonably well with the left shift of the voltage activation curve of the TRPM8 channel induced by cooling. This result is consistent with an earlier observation from deletion mutations that the last 72 amino acids of the TRPV1 *C* terminus affect channel activation [37]. On the other hand, the intracellular segment between the ankyrin-like repeat and S1 region of the channel has recently been proposed to serve as the thermal sensor for TRPV1 [38]. One potential solution to this controversy, offered in a recent stimulating article by Clapham and Miller [39], is that the high enthalpic and entropic changes associated with heat activation perhaps result from combined contributions of widely distributed sites. Cui *et al*. demonstrated that heat- and capsaicin-induced TRPV1 activations are structurally and mechanically distinct processes and that the pore turret is an indispensible channel structure involved in the heat activation pathway, but it is not a part of the capsaicin activation pathway [40]. Vanilloids interact at intracellular regions of TRPV1, as implied by the behavior of a membrane-impermeable charged capsaicin analog that is only effective when applied cytosolically [41]. Consistent with this observation, several intracellular molecular determinants of capsaicin binding have been identified. Two amino acid residues, Arg-114 in the *N* terminus and Glu-761 in the *C*-terminal domain, play a key role in ligand binding [42]. Amino acids Tyr-511 and Ser-512, located between the second and third transmembrane segments, are also critical for vanilloid binding and channel activation [43]; Thr-550 is involved in the structuring of vanilloid binding sites in rat and human TRPV1 channels [44]. The response of TRPV1 to vanilloids or heat is dynamically potentiated by extracellular protons [45]. Two glutamate residues located near the extracellular pore-forming region appear critical for the activity of the proton region. Glu-648, at the loop between the fifth and sixth transmembrane segments, is involved in direct activation of the channel at pH below 5.0, whereas Glu-600, located at the end of the fifth transmembrane segment, is important for the response of the channel to mildly acidic external conditions (pH 6.5). Nevertheless, Glu-600 neutralization gives rise to a constitutively active channel at 37 °C [37]. TRPV1 undergoes two types of desensitization on activation by vanilloids or protons: acute desensitization and tachyphylaxis or loss of sensitivity to repeated stimulation [46]. Receptor desensitization is believed to occur predominantly through a Ca^2+^-dependent process because it is largely abolished in the absence of Ca^2+^. An increase in the intracellular calcium concentration causes TRPV1 desensitization, and calmodulin (CaM), a ubiquitous calcium sensor, may mediate this effect. CaM interacts *in vitro* with isolated peptides from the TRPV1 *N*-terminal region in a Ca^2+^-dependent manner [47] and also binds to the TRPV1 *C*-terminal region in a Ca^2+^-independent manner [48].

## 3. The Physiological Role of TRPV1 in the Circulatory System and Kidneys

The role of TRPV1 receptors in circulation has been studied extensively. TRPV1 is one of the responsible receptors for the Bezold-Jarisch reflex, which causes hypotension, bradycardia, and apnea and occurs following the arterial injection of capsaicin [49]. TRPV1 is cardioprotective, mediating CGRP release in response to low pH and lactic acid in the guinea-pig heart [50]. Deletion of TRPV1 impairs post-ischemic recovery in the isolated perfused heart, exacerbates inflammation, and affects cardiac remodeling after myocardial infarction in mice [51]. Recent evidence using human samples has consolidated the beneficial cardiovascular effects of TRPV1 agonists such as capsaicinoids and capsinoids through ischemic preconditioning, release of neurotransmitters, and inhibition of platelet aggregation [52]. Exposure to cold is associated with oxidative stress [53] and cardiac dysfunction [54]. Both GSK3β inhibitors and TRPV1 agonists effectively rescue cold stress-induced contractile defects in mice. These results support a GSK3β-TRPV1-mediated mechanism in the regulation of cardiac contractile function and possible involvement of these molecules in the intracellular Ca^2+^ homeostasis under cold stress [55]. TRPV1 expression is observed in a high percentage of primary afferent neurons that project to cardiovascular and renal tissues [56]. It has been recently demonstrated that TRPV1 protein is present at high levels in the renal pelvis and exclusively regulates neuropeptide release from primary renal afferent nerves in response to mechanostimulation [57]. Moreover, TRPV1 protein is abundant in renal tubules of the medulla, although its function there is unknown [57]. The renal medulla contains nephron segments that are most susceptible to ischemic injury. We have recently obtained evidence that the TRPV1 agonists capsaicin, resiniferatoxin, and SA13353 (1-[2-(1-adamantyl)ethyl]-1-pentyl-3-[3-(4-pyridyl)propyl]urea) attenuate renal tumor necrosis factor (TNF)-a mRNA expression, increase renal interleukin (IL)-10 mRNA expression, and improve the condition of ischemia/reperfusion-induced renal injury in rats [58,59]. SA13353 is a novel TRPV1 agonist, which firstly cause pain by topical application like capsaicin, but has lower oral toxicity than capsaicin in rodents [60,61]. We speculate that the low distribution of SA13353 in brain after administration is one of the reasons of its low oral toxicity. The results from binding, enzyme inhibition, and functional assays show that SA13353 is an almost pure TRPV1 agonist [59]. CGRP is strongly coexpressed in many TRPV1-expressing nerve fibers, including the sensory fibers that innervate the dural vasculature [62], and it has been suggested that CGRP acts as a counterbalance to the development of hypertension [63]. The local effects of CGRP in the kidney are critical in preventing ischemia/reperfusion-induced acute renal injury in rats [64]. From the results using a TRPV1 antagonist and a CGRP antagonist, CGRP release by capsaicin-sensitive sensory neurons increases endothelial cell production of prostaglandin I_2_, which may contribute to the attenuation of the inflammatory response [64]. We suggest that the activation of capsaicin-sensitive afferent neurons by TRPV1 agonists and the resultant neuropeptide release may affect the inflammatory reactions after ischemia/reperfusion. TRPV1 agonists are therefore a new class of drugs important in renal pathology, particularly in ischemia/reperfusion-related kidney injury [65]. Further studies are required to establish a definite role of these agents in the protection from and treatment of renal diseases.

## 4. The Physiological Role of TRPV1 in Airway Inflammation and Disease

The afferent activities arising from sensory terminals in the lung and airways are primarily performed by branches of vagus nerves, which project to the nucleus tractus solitarius in the medulla. Among these sensory nerves, TRPV1 is predominantly expressed in non-myelinated afferent *C* fibers [66], which represent >75% of the afferent fibers in the pulmonary branch of the vagus nerve. One prominent anatomical feature of these sensory nerves is the axonal arborization of their endings. These endings either extend into the space between epithelial cells or form a network-like plexus immediately beneath the basement membrane of the epithelium [67,68], suggesting a role of these afferents in regulating airway responses to inhaled irritants [69]. When these TRPV1-expressing nerve endings are activated by inhaled irritants or endogenous TRPV1 activators, centrally mediated reflex responses are elicited, including reflex bronchoconstriction and mucus hypersecretion through the cholinergic pathway, accompanied by the sensation of airway irritation and the urge to cough. Sensory neuropeptides, especially tachykinins which are released by TRPV1 activation, are important for bronchoconstriction, protein extravasation and mucus secretion [70]. On the other hand, Somatostatin released from capsaicin-sensitive sensory nerves of the lung during endotoxin-induced murine pneumonitis inhibits inflammation and hyperresponsiveness, presumably through somatostatin receptor subtype 4 (sst_4_) [71]. Substantial upregulation of sst4 receptors during chronic inflammatory conditions in humans suggests the potential therapeutic significance of synthetic sst_4_ receptor agonists as novel tools for the treatment of inflammatory disease of the airway [72]. Synthetic sst_4_ receptor agonists inhibit acute and chronic airway inflammation and hyperreactivity in mice [73] and rats [74]. An increasing amount of evidence supports the hypothesis that the expression, activation, and modulation of TRPV1 in sensory neurons are integral components of the cough pathway, although the precise contribution of TRPV1 to human disease is yet to be determined [75,76,77]. In a bleomycin-induced scleroderma model in mice, TRPV1 activation and CGRP release exert protective actions against fibrosis [78]. The TRPV1 agonist capsaicin attenuates lung ischemia-reperfusion injury in rabbits [79]. We investigated the effects of orally administered TRPV1 agonists on leukocyte infiltration in LPS-induced acute lung injury and ovalbumin-induced allergic airway inflammation in rodents [80]. In LPS-induced lung injury, capsaicin and SA13353 attenuated neutrophil infiltration and the increase in TNF-a and cytokine-induced neutrophil chemoattractant (CINC)-1 levels. In allergic airway inflammation, SA13353 tended to inhibit leukocyte infiltration and attenuated the increase in IL-4 and IL-12p40. These results suggest that at least somatosensory TRPV1 may play an anti-inflammatory role in lung inflammation. Inducing the cough reflex and modifying airway inflammation may be important functions of TRPV1 in body homeostasis.

## 5. The Physiological Role of TRPV1 in Autoimmune Diseases

Current evidence for the role of TRPV1 in arthritis models is somewhat conflicting. Some groups have demonstrated that TRPV1 is involved in acute and chronic inflammation of the knee joint [18,19]. In contrast, other groups have shown that a TRPV1 agonist [81] and somatostatin [82] attenuate knee joint inflammation. Kissin *et al*. have explained that the anti-inflammatory effect of a TRPV1 agonist is due to desensitization of peripheral nerve in acute carrageenan-induced joint inflammation in rats [81]. However, there are no studies using the collagen-induced chronic arthritis model, one of the most important autoimmune models for human rheumatoid arthritis. We investigated the effects of the TRPV1 agonist SA13353 on the development of arthritis in collagen-induced arthritis in rats [60]. Post-onset treatment with SA13353 strongly reduced hind paw swelling and joint destruction associated with collagen-induced arthritis. The neuropeptides release was continually evoked after repeated SA13353 treatment. We believe that one of the mechanisms underlying the attenuation of arthritis development by SA13353 is inhibition of TNF-a production by inflammatory cells exposed to neuropeptides released from TRPV1-expressing afferent *C* fibers. In experimental autoimmune encephalomyelitis (EAE), another important autoimmune model, the agents activating cannabinoid and vanilloid receptors exhibit beneficial effects in rats [83]. We also investigated the effects of SA13353 on the development of EAE in mice [84]. SA13353 attenuated the clinical signs of EAE and associated histopathological changes, possibly by reducing inflammation in the spinal cord and cerebellum. We found that SA13353 also reduces the levels of a number of cytokines, including TNF-a, IL-1β, IL-12p40, IL-17, and interferon (IFN)-γ. In addition, SA13353 attenuated the increase in IL-17 production in splenocytes, implying that SA13353 inhibits the growth of Th17 cells and the development of EAE. The attenuation of TNF-a, IL-1β, and IFN-γ levels by SA13353 may also help inhibit EAE. More recently, it has been reported that TRPV1 channels modulate the synaptic effects of TNF-a and IL-1β in EAE [85]. TRPV1 channels play a protective role during the peak phase of EAE by reducing the severity of EAE and limiting the synaptic effects of TNF-a. In contrast, in the chronic phase, these channels play a proinflammatory role, being necessary for IL-1β-mediated inhibition of the GABAergic transmission. Myeloid-derived suppressor cells (MDSCs) are newly identified cells of the myeloid lineage that co-express CD11b and Gr-1 antigens [86]. These cells possess some potent suppressive functions and regulate inflammatory responses [87,88]. MDSCs play a critical role in attenuating acute inflammation in the liver, and activation of TRPV1 receptors, which trigger MDSCs, may constitute a novel therapeutic modality to treat inflammatory diseases [89]. Further studies are necessary to clarify the role of TRPV1 in autoimmune diseases.

## 6. The Physiological Role of TRPV1 in Eye Diseases

The cornea is densely innervated with sensory nerve fibers whose cell bodies reside in the ipsilateral trigeminal ganglion. Nerve bundles enter the peripheral corneal stroma in the middle, divide dichotomously as they extend toward the center of the cornea, branch into a sub-basal plexus between the stromal and epithelial layers, and terminate between the epithelial cells of the basal layer and more superficial layers [90,91]. Nerve terminals in the corneal epithelium are unmyelinated and express TRPV1 frequently [92,93]. This is consistent with the acute sensitivity of the cornea to noxious stimuli. Capsaicin-induced eye-wipe response is one of the important responses to TRPV1 agonists [94]. On the other hand, topical resiniferatoxin, a potent TRPV1 agonist, may be a valuable tool for managing post-surgical eye pain by inactivating TRPV1-expressing nerve terminals through excessive Ca^2+^ influx, with many clear advantages over local anesthetics, NSAIDs, and opiates [95]. TRPV channels also mediate temperature sensing in human corneal endothelial cells [96]. It has been shown that capsaicin induces cell death in the inner retina of preweaning rats [97]; however, it remains uncertain whether this effect is due to TRPV1 activation or mitochondrial impairment. These data indicate that TRPV receptors may also play a role in developmental processes. TRPV1 is detectable in rat retinas in the initial steps of development and in adult retinas, whereas the TRPV2 receptor has been found only in the late phases of retinal development [98]. TRPV1 channels are involved in the control of early apoptosis during retinal development, and mitogen-activated protein kinase signaling may be involved in this process [99]. Moreover, TRPV1-mediated signaling may be important for neuronal differentiation and synaptic maturation in the retina. Elevated intraocular pressure (IOP) is the leading risk factor for the degeneration of retinal ganglion cells (RGCs) and their axons during traumatic injury and in chronic disease, particularly glaucoma [100,101]. RCGs express TRPV1 mRNA, with abundant TRPV1 protein localization in the cell body and axon. TRPV1 activation can induce *in vitro* RGC apoptosis in the absence of other insults, most likely through the influx of extracellular Ca^2+^ [102]. On the other hand, the mouse retina expresses mRNA and protein for the polymodal TRPV4 cation channel known to mediate osmotransduction and mechanotransduction [103]. TRPV4 is expressed in RGCs, and its activation mediates the response to membrane stretch, leading to the elevation of Ca^2+^ levels and augmented excitability. Excessive Ca^2+^ influx through TRPV4 predisposes RGCs to the activation of Ca^2+^-dependent proapoptotic signaling pathways, indicating that TRPV4 is a component of the response mechanism to the pathological elevation of IOP. The TRPV channel important for RGC apoptosis remains controversial. Some recent studies show that neuronal cell death induced by ischemic-reperfusion or NMDA can be reduced by capsaicin in the rat retina [104,105]. In an NMDA model, CGRP also reduced neuronal cell death in the retina. In addition, the use of potent somatostatin analogs may be considered safe for stopping the progression from pre-proliferative to proliferative diabetic retinopathy [106].

## 7. Therapeutic Potential of TRPV1 Agonists

**Figure 1:**
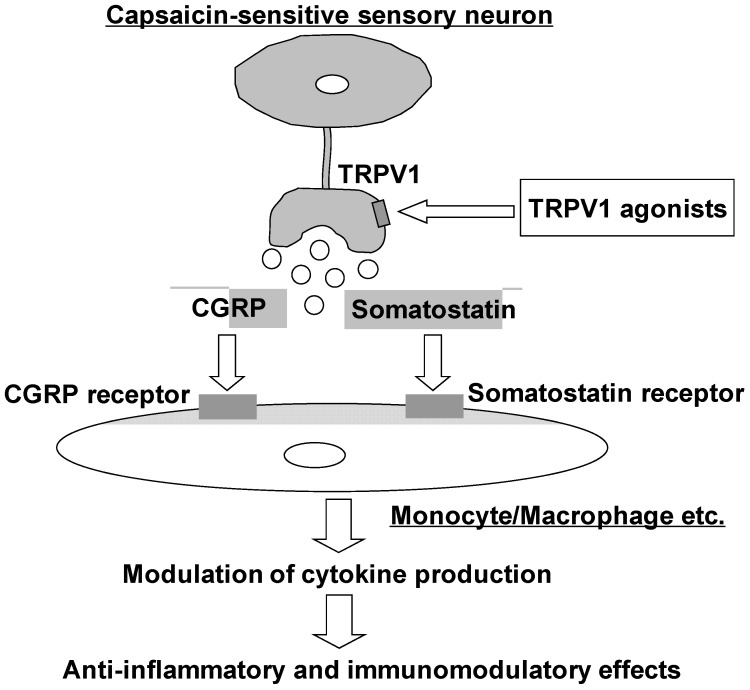
Potential mechanism of the anti-inflammatory and immunomodulatory effects of TRPV1 agonists.

As mentioned above, TRPV1 agonists can act as anti-inflammatory and immunomodulatory agents in certain inflammatory diseases. We believe that TRPV1 agonists modulate cytokine production through neuropeptide release from afferent *C* fibers and show anti-inflammatory and immuno-modulatory effects. With numerous studies suggesting that TRPV1 agonistic effects are associated with pain and inflammation [107], many pharmaceutical companies have developed TRPV1 antagonists [108]. However, many TRPV1 antagonists were withdrawn because of their potential to cause hyperthermia and the danger of affecting the important physiological functions of TRPV1 in the peripheral and central nervous system [109]. Because antagonist-induced hyperthermia is an on-target effect and a hurdle for the development of TRPV1 antagonists as therapeutics, future clinical trials of this class of molecules may include the following: (i) a preference to develop TRPV1 antagonists with shorter half-lives; (ii) co-dosing with antipyretic agents such as acetaminophen; and/or (iii) exclusion of patients susceptible to frequent pyrexia [110]. Other efforts have targeted the optimization of TRPV1 agonist-based therapies, primarily to inactivate nociceptive nerve fibers [11]. Data from several clinical studies of TRPV1 agonists indicate their potential efficacy in pain relief associated with postherpetic neuralgia, diabetic neuropathy, osteoarthritis, bunionectomy, Morton’s neuroma, and post-surgical pain following orthopedic surgery. In some cases, these same concepts may be applied to the cardiovascular, respiratory, *etc*. systems. The use of TRPV1 agonists for treating diabetic peripheral neuropathy seem counter-intuitive since activation of these channels in peripheral nerves lead to damage of killing of these nerves. However, low dose systemic application of SA13353 tended to inhibit the reduction of motor nerve conduction velocity in streptozotocin-induced diabetic rats [111]. Clinical studies of topical capsaicin have often suggested beneficial effects in reducing pain associated with diabetic neuropathy, osteoarthritis and psoriasis [112]. On the other hand, activation of TRPV1 mediates temporary hearing loss by initiating an inflammatory process in the cochlea via activation of NOX3 NADPH oxidase and signal transducer and activator of transcription 1 (STAT1) [113]. Table 1 shows the current clinical trial status of TRPV1 and somatostatin modulators developed by different drug discovery companies for the treatment of pain and inflammation. We have identified another potential application of TRPV1 agonists in the modulation of immune responses at doses lower than those required for nerve inactivation. Some of the effects of TRPV1 agonists observed to date, which are currently attributed to the inactivation of nociceptive nerve fibers, may be caused by their immunomodulatory effects. If afferent *C* fibers indeed release a critical mediator associated with anti-inflammatory and immunomodulatory effects, they would serve as a target for the treatment of autoimmune diseases (Figure 1). Under chronic polyneuropathic conditions, it has been reported that TRPV1 receptor can initiate antinociceptive counter-regulatory mechanisms possibly mediated by continually somatostatin released from sensory neurons [114]. We believe that such a finding justifies the evaluation of the value of TRPV1, CGRP, and somatostatin modulators for clinical applications as analgesic, anti-inflammatory, and immunomodulatory agents.

**Table 1:** Current clinical trial status of TRPV1 and somatostatin modulators developed by different drug discovery companies.

Compound name	Brand name	Molecular mechanism	Company	Route of administration	Indication	Stage
Capsaicin	Qutenza	TRPV1 agonist	NeurogesX	Topical patchesTopical cream	Neuropathic pain	Launched
Zucapsaicin	Zuacta	TRPV1 agonist	Winston Laboratories	Topical cream	Osteoarthritis	Launched
SB-705498	―	TRPV1 antagonist	GlaxoSmithKline	Intranasal	Rhinitis	Phase II
PAC-14028	―	TRPV1 antagonist	AmorePacific	Oral	Atopic dermatitis	Phase I
SYL-1001	―	TRPV1 expression inhibitor	Sylentis	Ophthalmic	Dry eyePain	Phase I
DG-3173	―	Somatostatin analog	Evotec	Subcutaneous	Diabetic retinopathy	Phase I

## 8. Conclusions

TRPV1 agonists and neuropeptides released as a result of TRPV1 activation might act *in vivo* as anti-inflammatory and immunomodulatory agents in certain inflammatory diseases. Further studies are therefore necessary to clarify the role of TRPV1 and neuropeptides in inflammation and autoimmune diseases.
